# Trigger finger genetics and its tendon pathology

**DOI:** 10.1186/1753-6561-9-S3-A64

**Published:** 2015-05-19

**Authors:** Anna-Carin Lundin

**Affiliations:** 1 Department of Hand and Plastic Surgery, University Hospital, 581 85 Linkoping, Sweden

## 

The pathogenesis of trigger finger has generally been ascribed to primary changes in the first annular ligament. In contrast, we recently found histological changes in the tendons, similar to the findings in Achilles tendinosis. When we looked further for more similarities we found differences in gene expression in the trigger finger tendons in comparison to normal tendons, in a pattern similar to what is published for Achilles tendinosis. We also found SNP (Single Nucleotide Polymorphism) pattern difference with similarities with what is published for Achilles tendinosis.

